# Empathic Fear Responses in Mice Are Triggered by Recognition of a Shared Experience

**DOI:** 10.1371/journal.pone.0074609

**Published:** 2013-09-18

**Authors:** Jeff Sanders, Mark Mayford, Dilip Jeste

**Affiliations:** 1 Sam and Rose Stein Institute for Research on Aging, and Department of Psychiatry, University of California San Diego, La Jolla, California, United States of America; 2 Dorris Center for Neuroscience, The Scripps Research Institute, La Jolla, California, United States of America; 3 Department of Neurosciences, University of California San Diego, La Jolla, California, United States of America; Peking University, China

## Abstract

Empathy is an important psychological capacity that involves the ability to recognize and share emotions with others. In humans, empathy for others is facilitated by having had a similar prior experience. It increases with the intensity of distress that observers believe is occurring to others, and is associated with acute emotional responses to witnessing others’ distress. We sought to develop a relatively simple and fast mouse model of human empathy that resembled these characteristics. We modeled empathy by measuring the freezing of observer mice to observing the footshock of a subject mouse. Observer mice froze to subject footshocks only when they had a similar shock experience 24 hours earlier. Moreover, this freezing increased with the number of footshocks given to the subject and it was accentuated within seconds after footshock delivery. Freezing was not seen in naïve observers or in experienced observers that observed a subject who was spared footshock. Observers did not freeze to a subject’s footshock when they had experienced a swim stress 24 hours prior, demonstrating a specific effect for shared experience, as opposed to a generalized stressor in eliciting observer mouse freezing. We propose that this two-day experimental protocol resembles many aspects of human empathy in a mouse model that is amenable to transgenic analysis of neural substrates for empathy and its impairment in certain clinical disorders.

## Introduction

Empathy is an important capacity that involves both the recognition of other’s mental states and the generation of an appropriate emotional response to these states [1]. Pathological conditions such as antisocial personality disorder (ASPD) and psychopathy, which are characterized by the callous disregard for the emotions and rights of others, highlight the importance of empathy to psychosocial functioning [2], [3]. Deficits in empathy can, in extreme cases, lead to acts of severe aggression toward others [4], [5], and have also emerged as an important social cognitive deficit in schizophrenia [6]. Although these observations underscore the importance of understanding the origins of empathy, the neurobiological systems that subserve this capacity are poorly understood. This understanding has been delayed, in part, by the inherent limitations of research in human subjects, pointing to a need for animal models for empathy to study its underlying brain mechanisms.

Empathy can be further defined in terms of both cognitive empathy, which refers to the ability to understand the viewpoint of another, and emotional empathy, which refers to the ability to recognize and share emotions with another [1]. Emotional empathy, in particular, is impaired across a wide range of psychiatric conditions. Psychopathy, for instance, is characterized by specific impairments in emotional empathy [3] and by specific deficits in recognizing emotions and distress in others [7], [8]. Emotional empathy is also disrupted in alcohol dependence and frontotemporal dementia and in some sex offenders [9]–[11]. Although the widely observed impairments in emotional empathy point to the clinical importance of understanding this ability, few animal models have historically been proposed for its study. Furthermore, animal models for ASPD and psychopathy have largely focused on studying aggressive behaviors in rodents [12], and have not modeled emotional empathy and sensitivity to others’ distress, the deficits that are considered central to the pathogenesis of these disorders [3], [8], [13].

Recent experiments [14]–[20] have built upon seminal studies in rats [21], and propose that emotional empathy may be studied in the behavior of rodents in response to observing conspecific distress. Emotional responses to conspecific distress have also been found in chickens [22] as well as pigeons [23], suggesting that a basis for emotional empathy may be widely conserved across species. Moreover, recent investigations show that rats are not only sensitive to conspecific distress, but that rats also act to relieve this distress [24]. This suggests that a rodent’s capacity to recognize and feel others’ distress may provide the basis for pro-social helping behavior that is empathically motivated [24].

The further development of mouse models of empathy would be especially useful for applying modern transgenic methods to explore the neural basis for such behaviors. While mice might be expected to behave in a similar way to rats, behavioral paradigms in rats can be difficult to extend to mice and may require various modifications [25], [26]. Moreover, species differences between mice and rats have been reported in socially modulated fear behaviors used to model empathy [27], [28].

Optimally, a mouse model of empathy would hold face validity with many characteristics of human empathy. For instance, while some aspects of empathy appear to be innate [29], several others, including the ability to generate emotionally empathic responses to others’ distress [30], are promoted by sharing similar aversive life experiences [30]–[35]. Furthermore, human data show that empathy-related measures increase with intensity of the aversive experience that observers believe is occurring to others [34], [36]–[38]. Finally, perceiving others’ distress is associated with acute physiological indices of emotional response that occur within seconds of observation [39].

We sought to develop a relatively simple and fast mouse model of human empathy. To model the above mentioned characteristics of human empathy in mice, we studied the freezing behavior of an observer mouse to multiple footshocks given to a subject mouse. We also measured the timecourse of the observer’s fear responses post-footshock. We hypothesized that observer mice would freeze to observing subject footshock when they had specifically experienced a similar footshock experience, but not when they had experienced a preceding stressor that was dissimilar. We further hypothesized that observer freezing would increase with the number of shocks delivered to subjects, and that it would be accentuated immediately after the observation of footshock. Since animal models of empathy vary widely in their time to perform [14], [15], [18], [19], [40], [41], sometimes involving habituation procedures and taking several days to complete [14], [19], [27], [40], we sought to develop a method that is completed over a relatively short time frame. We also sought to develop a method that delivers a relatively low amount of aversive stimuli to mice than has been reported in other empathy models [18], [19], [41]. In this paper we discuss a two-day experimental paradigm that uncovers several novel mouse behaviors which hold face validity with features of human empathy.

## Methods

### Animals

C57BL/6J mice were used from in house breedings. Mice were kept in a temperature controlled room and maintained on a 12 h/12 h light-dark cycle with *ad libitum* access to food and water. Mice were socially housed as 2–4 mice per cage from weaning until experiments. Cagemates were housed together for at least 5 weeks. The study was carried out in strict accordance with the recommendations in the Guide for the Care and Use of Laboratory Animals of the National Institutes of Health. The protocol was approved by the The Scripps Research Institute guidelines for the humane care and use of laboratory animals (IACUC Protocol 09-0071-2) and all efforts were made to minimize suffering. We used male and female mice between 2–6 months of age.

### Behavioral Protocol

Our experimental protocol consisted of an ‘observer’ mouse whose freezing was recorded while it witnessed the experience of a ‘subject’ mouse. Our protocol consisted of four experimental groups: (1) SH_obs_ = observers that received footshocks in context A on day 1, and then observed footshocks given to a subject in context B on day 2, n = 15 (9 males/6 females). (2) SHN_obs_ = observers that received footshocks in context A on day 1, and then observed a subject in context B, where footshocks were not delivered on day 2, n = 15 (8 males/7 females). (3) SW_obs_ = observers that underwent forced swim stress on day 1, and then observed footshocks given to a subject in context B on day 2, n = 13 (6 males/7 females). (4) Naïve = observers that remained in their homecage on day 1, and then observed footshocks given to a subject in context B on day 2, n = 17 (10 males/7 females). Mice from the SH_obs_ and SHN_obs_ groups were excluded from the study if they did not demonstrate adequate fear learning on day 1. Observers and subjects in each group were always of the same sex.

On day 1, SH_obs_, and SHN_obs_ received footshocks in context A, which consisted of a fear conditioning chamber (30-cm length×24-cm width) with a white curved wall insert (Med Associates Inc.). This chamber was illuminated with a red light and scented with mint. On the bottom of the chamber was a grid floor that delivered footshocks (FreezeFrame). Footshocks were delivered within a contextual fear conditioning session consisting of 120 s of free exploration followed by six non-signaled foot shocks (duration 1 s, intensity 0.7 mA) with an interstimulus interval of 15 s for a total duration of 216 s. On day 1 SW_obs_ underwent swim stress for 2 min by being placed in a plastic cylinder with water at a temperature of 22C and a depth ∼15 cm so that they could not touch the bottom. Following exposure to these experiences, mice from these experimental groups were returned to their homecage.

On day 2 SH_obs_, SHN_obs_, SW_obs_ and Naïve observers were then placed in context B. Context B consisted of a lemon-scented fear conditioning chamber (30-cm length×24-cm width) that was illuminated with white light and consisted of a checkerboard wall and a grid floor upon which a subject mouse was placed for the entire duration of the session. The subject’s experience in context B was witnessed by an observer within a smaller transparent plastic container situated within context B whose floor was covered with sani-chips (Allentown caging, base: 20-cm length×12-cm width, top: 22-cm length×14-cm width). For SH_obs_, SW_obs_ and Naïve experimental groups the session in context B consisted of 120 s of free exploration during which time we first measured the baseline freezing of the observer. This was followed by its observation of six non-signaled foot shocks delivered to the subject on the grid floor (duration 1 s, intensity 0.7 mA). During interstimulus intervals of 15 s we measured the freezing of the observer to witnessing subject footshock. The total session duration was 216 s. For the SHN_obs_ experimental group a similar experimental protocol was used, however footshock was not delivered to the subject.

We performed additional experiments where SH_obs_ observed a subject in context B on day 2 who was protected from the delivered footshocks by a single layer of clear Saran Wrap (S.C. Johnson and Son, Inc.) placed on top of the shock grid. This was done to rule out potential cues produced by the equipment that could signal shock to the SH_obs_ mouse during footshock delivery (SH_obs_-Block) n = 8 (4 males/4 females).

### Behavioral Scoring and Analysis

We video recorded the freezing behavior of our observer mice and used an automated system for measuring their freezing to witnessing the experience of subjects (FreezeView). On day 1, the fear acquisition of SH_obs_ and SHN_obs_ groups in context A was measured in 15 s bins after each of the 6 non-signaled foot shocks. On day 2 a cardboard mask was placed on top of the fear conditioning chambers in order to selectively measure the freezing of the observer mouse in context B and exclude detection of the subject’s behavior. For SH_obs_, SHN_obs_, SW_obs_ and Naïve groups percent freezing was measured in 3, 40 s bins during the 120 s of exploration in context B to obtain a baseline freezing measurement. SH_obs_, SW_obs_ and Naïve percent freezing was then measured in 15 s bins after each of the 6 non-signaled foot shocks delivered to the subject. In the SHN_obs_ group percent freezing was measured in the 15 s bins after the 6, 1 s shocks delivered to the subject in the other experimental groups. Further analysis of SH_obs_ freezing data quantified freezing in the 5 s after footshock observation relative to the remaining 10 s.

### Statistical Analysis

Fear acquisition in context A and the freezing of observers in context B were analyzed with a repeated measures ANOVA, where we analyzed experimental group and sex as between group factors and time interval in the context as a within group factor. This was followed by Fisher post-hoc analysis. Statistical comparison of total SH_obs_ freezing in the 5 s interval immediately after cagemate footshock compared to the remaining 10 s interval was performed with a Student’s t-test.

## Results

**Figure 1:**
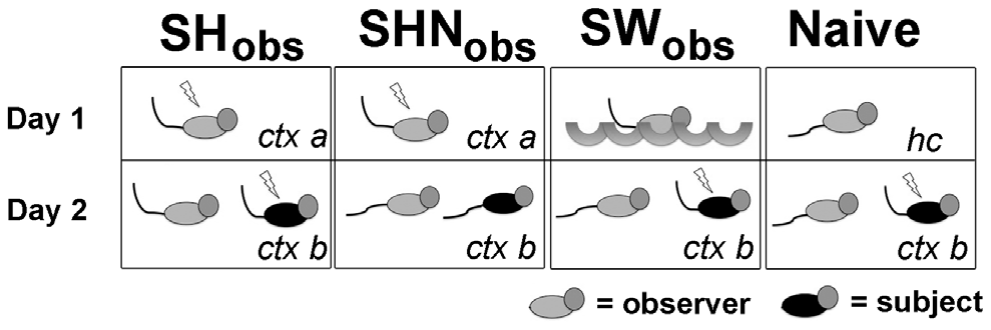
Diagram of Experimental Design. We designed a two-day experimental protocol where mice were randomly assigned to one of four experimental groups: (1) SH_obs_ = observers that received footshocks in context A on day 1, and then witnessed footshocks given to a subject in context B on day 2, n = 15. (2) SHN_obs_ = observers that received footshocks in context A on day 1, and then witnessed a subject in context B, where footshocks were not delivered on day 2, n = 15. (3) SW_obs_ = observers that underwent forced swim stress on day 1, and then witnessed footshocks given to a subject in context B on day 2, n = 13. (4) Naïve = observers that remained in their homecage on day 1, and that witnessed footshocks given to a subject in context B on day 2, n = 17.

We first investigated how prior footshocks modified the freezing of observers to witnessing subjects having a similar experience. Observers were contextually fear conditioned in context A on day 1. On day 2, one group of observers (SH_obs_) was placed in context B where they witnessed the contextual fear conditioning of subjects in this context (Figure 1). A second group of these observers (SHN_obs_) were placed in context B where they witnessed subjects for a similar duration of time within this context, but footshock was not delivered. This group was included as a control for the possible recognition of contextual cues in context B that could influence freezing independent of observing the subject’s footshocks. We controlled for non-specific effects of a prior stressor by exposing a separate group of observers (SW_obs_) to forced swim on day 1, and placed them in context B on day 2 where we measured their freezing to witnessing subject footshock in context B. We compared the freezing of these experimental groups to naïve mice that remained in their homecage during day 1 and who were then placed in context B on day 2 where they observed subject footshock (Naïve) (Figure 1). In our initial experiments the observers and subjects were cagemates.

**Figure 2:**
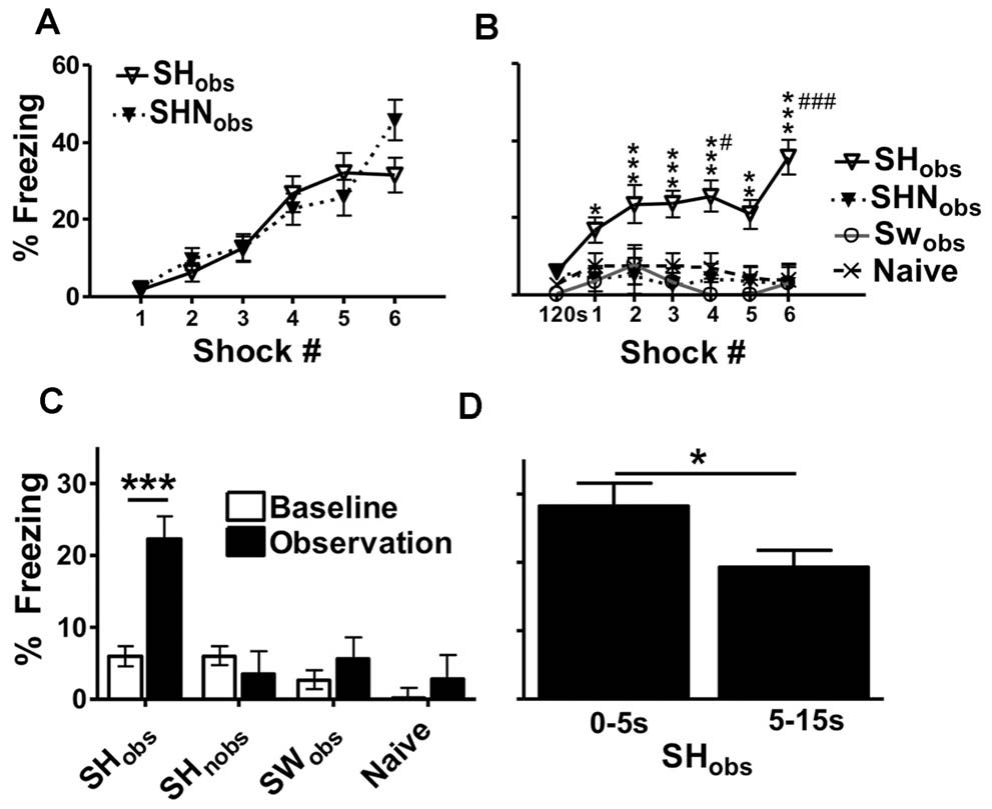
Freezing is Triggered by Recognition of a Shared Experience. **A)** SH_obs_ and SHN_obs_ showed similar acquisition of contextual fear learning. **B)** Despite similar fear learning to SHN_obs_, SH_obs_ uniquely froze over baseline after each of the footshocks delivered to cagemates. Significant differences were found for freezing that was above baseline context B levels. SH_obs_ froze higher after observing footshock 4 compared to footshock 1, and after observing footshock 6 compared to each of the preceding footshocks (#p<0.05, ###p<0.001). **C)** SH_obs_ had higher total observational freezing scores compared to baseline. The total observational freezing for SH_obs_ was also higher than total observational freezing of each of the other experimental groups. Within each experimental group there were no differences in the freezing of sexes in context A or context B. **D)** SH_obs_ freezing during the collected 5 s interval immediately after each footshock and compared to total freezing during the remaining 10 s after each footshock. SH_obs_ froze more in the 5 s interval immediately after witnessing cagemate footshock, with lower freezing during the remaining 10 s. In these experiments observers and subjects were cagemates. Data are mean freezing scores +/− S.E.M (n = 13–17 mice per group) (*p<0.05, **p<0.01, ***p<0.001).

On day 1, SH_obs_ and SHN_obs_ equally acquired fear learning in context A, (main effect of trial; F(5,130) = 29.6, p<0.001) (Figure 2A). On day 2, all experimental groups showed low levels of freezing during a 120 s baseline period in context B (∼0–6%), without differences between the groups. However, SH_obs_ behaved significantly different from all other groups during the subsequent period during which they observed cagemates receiving footshocks (main effect for group (F(3,52) = 12.1, p<0.001). After the first presentation of a shock to the subject, SH_obs_ froze significantly more than at baseline (p<0.05). Freezing increased further with consecutive shocks across the subsequent 6 time bins (F(6,312) = 2.97, p<0.05), reaching up to 40%. Thus, freezing levels were higher after observing footshock 4 compared to footshock 1 (p<0.05), and after observing footshock 6 compared to each of the preceding footshocks (p<0.05 to p<0.001, Figure 2B).

In contrast, none of the other groups showed freezing levels different from baseline at any of the time points (group × time interval interaction (F(18,312) = 2.61, p<0.001, Figure 2B). Importantly, observing a subject receive footshock did not cause freezing on its own. Even swim stress experienced 24 hrs before the test was not sufficient to induce the same freezing during the observation of a subject getting shocked. Furthermore, fear memory was not generalized from context A to B, since freezing levels in the SHN_obs_ did not differ from baseline values (Figure 2B).

In humans, empathy is reported to be greater in females than in males [42], [43]. Therefore we studied whether their were differences in the freezing of female versus male mice. Within each experimental group, however, there were no differences in the freezing of sexes in context A, (F(5,130) = 1.3, p = 0.3), or in context B, (group × sex (F(3,42) = 2.6 p = 0.06, group × time interval × sex (F(18.312) = 0.42918, p = 0.98). Therefore we pooled male and female mice within our studies.

Observational freezing in SH_obs_ was further emphasized in the comparison of baseline and total observational freezing across groups (main effect of group: F(3,52) = 10.3, p<0.001). Baseline and total observational freezing differed significantly in this experiment (effect of time interval in context B (baseline versus observation period): F(1,52) = 18.8, p<0.001), but this was largely due to the large increase in freezing seen in the SH_obs_ (interaction between group and time interval in context B: F(3,52) = 12.0, p<0.001). The total observational freezing for SH_obs_ was >20% and was uniquely elevated above baseline (p<0.001), compared to the other experimental groups where observational freezing was ∼2–5% (Figure 2C). The total observational freezing for SH_obs_ was also higher than the total observational freezing of each of the other experimental groups (p<0.001).

To examine the distribution of freezing within the intervals between footshock, we separated SH_obs_ freezing during the first 5 s immediately after each footshock from the remaining 10 s inter-shock interval and compared the two. SH_obs_ froze more in the 5 s interval immediately after witnessing subject footshock, with lower freezing during the remaining 10 s (p<0.05) (Figure 2D).

**Figure 3:**
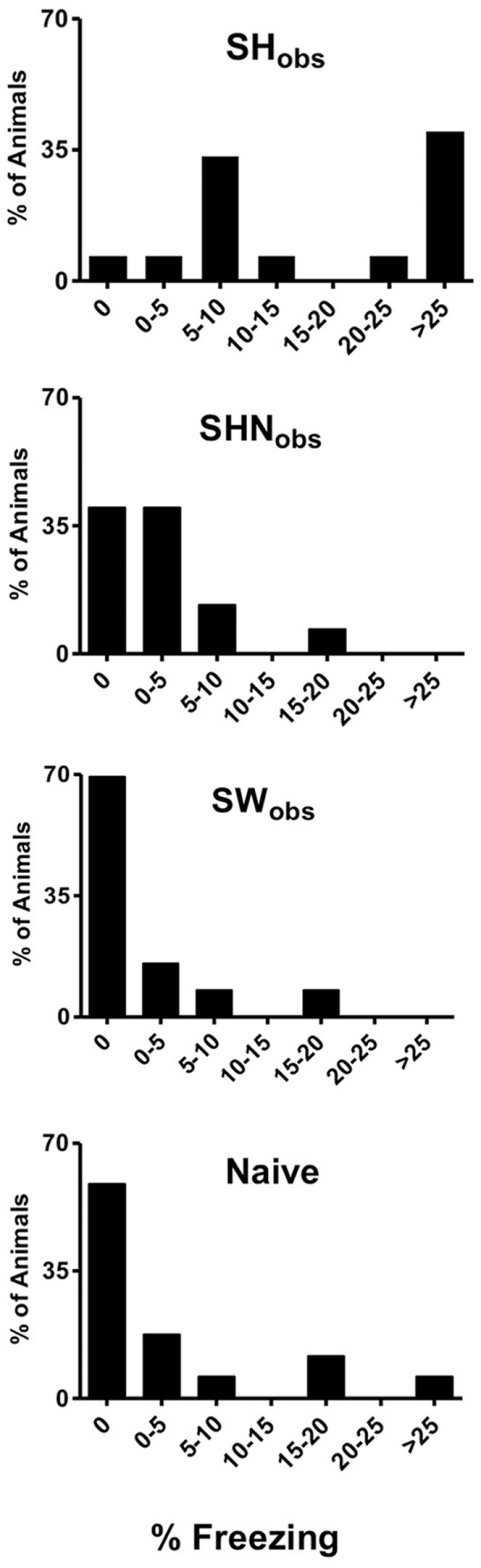
Distribution of Freezing Levels for SH_obs,_ SHN_obs_, SW_obs_ and Naïve Groups. Many SH_obs_ froze greater than 25%. SHN_obs_, SW_obs_ and Naïve groups showed a more animals with freezing scores less than 5% and very few mice freezing greater than 25%. Data are % of animals freezing within a defined range.

We next analyzed the freezing distribution of SH_obs,_ SHN_obs_, SW_obs_ and Naïve individuals. The majority of SH_obs_ mice displayed freezing levels less than 20%. However, there was substantial variability, with many of these mice freezing greater than 25% (Figure 3). In contrast, the freezing distribution of SHN_obs_, SW_obs_ and Naïve groups showed many animals with freezing scores less than 5% with very few mice freezing greater than 25% (Figure 3). The freezing levels of SH_obs_ therefore demonstrate considerable heterogeneity, suggesting that variations between individual mice could be studied to understand the factors contributing to observational freezing behavior.

**Figure 4:**
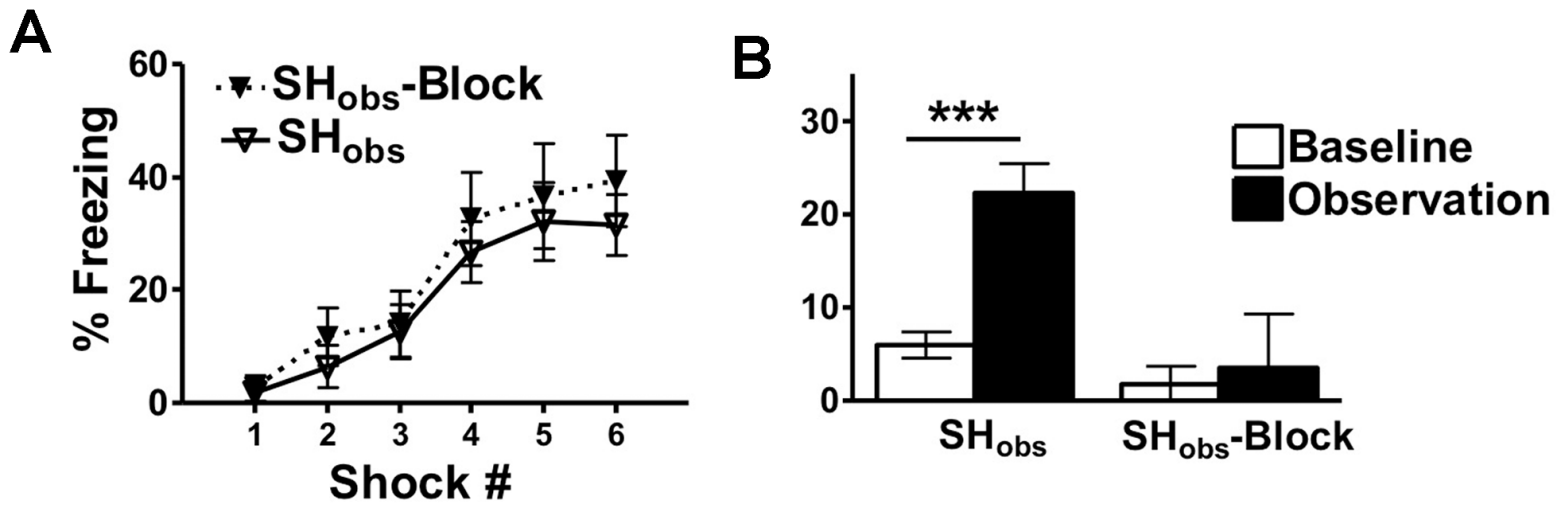
Protecting Subjects from Footshock Eliminates Freezing in SH_obs_. A) SH_obs_ and SH_obs_-Block showed similar acquisition of contextual fear learning. B) Observational freezing is eliminated **in** SH_obs_-Block. In these experiments observers and subjects were cagemates. SH_obs_ = observers that received footshocks in context A on day 1, and then witnessed footshocks given to a subject in context B on day 2. SH_obs_-Block = observers that received footshocks in context A on day 1, and then witnessed a subject in context B on day 2 who was protected from delivered footshocks. Data are mean freezing scores +/− S.E.M (n = 8–15 mice per group) (***p<0.001). Within each experimental group there were no differences in the freezing of sexes in context A or context B.

As an added control we performed experiments where SH_obs_ observed a subject in context B where shocks were delivered, but where the subject was protected from shock by a thin barrier placed upon the shock grid (SH_obs_-Block). This was performed to rule out any cues produced from the equipment during shock delivery that could have served as a CS to the SH_obs_ mouse. On day 1 SH_obs_ and SH_obs_-Block equally acquired fear learning in context A F(5,95) = 20.6, p<0.001 (Figure 4A). On day 2 there were significant differences in the freezing of SH_obs_ and SH_obs_-Block (main effect of group: F(1,19) = 10.9, p<0.05). Although there were low levels of baseline freezing in context B that did not differ between the two groups, SH_obs_ behaved significantly different during the observation period (effect of time interval in context B (baseline versus observation period): F(1,19) = 13.4, p<0.01). Upon witnessing the footshock of the subject, SH_obs_ froze significantly more than at baseline (p<0.001) with total SH_obs_ freezing increasing to >20%. In contrast, SH_obs_-Block did not show observational freezing levels different from baseline (interaction between group and time interval in context B F(1,19) = 8.97, p<0.05) (Figure 4B). Thus, artifactual freezing in response to cues produced from the equipment that reminded the SHobs mice of the experience the previous day was not a likely cause for SH_obs_ freezing.

Fear memory undergoes a time-dependent consolidation with distinctive properties characteristic of short-term memory versus consolidated long-term memories [44]. We examined whether SH_obs_ freezing was unique to a 24 hr timepoint post-fear conditioning, or whether it could also be triggered at an earlier timepoint, when little memory consolidation had taken place. To test this, we examined whether SH_obs_ freezing could be observed as short as 30 minutes after fear conditioning (SH_obs_−_30 min_).

**Figure 5:**
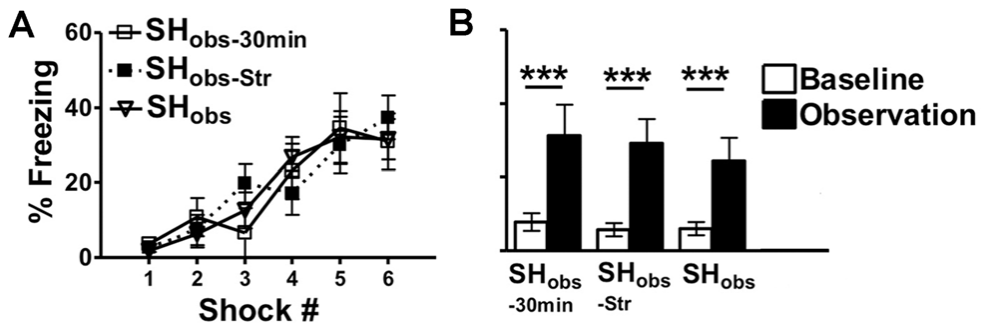
SH_obs_ Freezing Occurs as Early as 30 min Post-Observer Footshock and Occurs Between Cagemates and Strangers. A) On day 1, SH_obs_, SH_obs_-_Str_ and SH_obs_−_30 min_ revealed a significant acquisition of fear learning in context A without differences between groups. B) On day 2, SH_obs_, SH_obs_-_Str_ and SH_obs_−_30 min_ showed similar levels of low baseline freezing and a similar levels of observational freezing. SH_obs_−_30 min_ = observers that received footshocks in context A, and then witnessed footshocks given to cagemate in context B 30 min later. SH_obs-Str_ = observers that received footshocks in context A on day 1, and then witnessed footshocks given to a non-cagemate in context B on day 2. SH_obs_ = observers that received footshocks in context A, and then witnessed footshocks given to cagemate in context B 24 h later. Data are mean freezing scores +/− S.E.M (n = 8–15 mice per group). (***p<0.001). Within each experimental group there were no differences in the freezing of sexes in context A or context B.

On day 1, SH_obs_ and SH_obs_−_30 min_ equally acquired fear learning in context A, (main effect of trial; F(5,95) = 18.0, p<0.001) (Figure 5A). On day 2, baseline and observational freezing differed significantly (effect of time interval in context B (baseline versus observation period): F(1,19) = 22.5, p<0.001. However, there were not any differences in the freezing levels of SH_obs_ and SH_obs_−_30 min_. These groups had similar levels of low baseline freezing in context B and similar levels of observational freezing that were elevated above their baseline values (p<0.001) (Figure 5B). Therefore, SH_obs_ freezing may be detected as rapidly as 30 min, within a period of minimal fear memory consolidation, as well as 24 hours after the fear conditioning of the observer.

In humans, empathy towards others is not necessarily selective for familiar others and may be seen between strangers as well close relations [45]. In our experiments, up until this point, the observers and subjects were cagemates. We next examined the freezing of SH_obs_ mice who witnessed the fear conditioning of non-cagemates who were therefore strangers to the fear conditioned animal (SH_obs_-_Str_). On day 1, SH_obs_ and SH_obs_-_Str_ equally acquired fear learning in context A, (main effect of trial; F(5,120) = 23.9, p<0.001) (Figure 5A). On day 2, baseline and observational freezing differed significantly (effect of time interval in context B (baseline versus observation period): F(1,24) = 48.6, p<0.001. However, there were not any differences in the observational freezing levels of SH_obs_ and SH_obs_-_Str_. These groups had similar levels of low baseline freezing in context B and similar levels of observational freezing that were elevated above their baseline values (p<0.001) (Figure 5B). Therefore, SH_obs_ freezing is a robust behavior that may be obtained when mice observe the footshock of cagemate or non-cagemate subjects.

## Discussion

Our studies found that observer mice froze to subject footshock in context B only when they had a similar experience in context A 24 hours earlier. Since this freezing was not seen during the 120 s baseline period in context B, or during an extended period of observation in context B in SHN_obs_, it excluded the recognition of contextual cues as a source of this freezing. Our additional controls, which we describe in detail below, established SH_obs_ freezing as specifically triggered by their recognition of a shared experience with subject mice.

While we found that observer mice froze to subject footshocks only when they have had a similar shock experience, Jeon and colleagues found that naïve observer mice will freeze to witnessing footshock [41]. This difference may be due to the higher level of aversive stimuli delivered to subjects by Jeon et al. [41] than in our studies. We delivered fewer footshocks to subjects, with each of shorter duration, lower amplitude and at more widely spaced intervals (ie. 6,1 s, 0.7 mA, footshocks delivered every 15 s) than did Jeon et al. [41]. Collectively, these data suggest that in naïve observer mice a high level of aversive stimuli occurring to subjects is required to obtain freezing. However, if observers have had a prior footshock experience themselves, they respond to lower levels of aversive stimuli occurring to subjects.

Consistent with our data in mice, findings in rats and pigeons have demonstrated that a prior shock experience potentiates fear responses to witnessing the shock of another animal [14], [21], [23]. It is possible that observer freezing in these reports, and SH_obs_ freezing in our data, was attributable to a generally heightened state of anxiety as a result of having had a prior stressful experience. This would lead to a generalized tendency to subsequently show fear, irrespective of whether observers were witnessing a similar experience to their own occurring to subjects. In our experiments we controlled for the non-specific effects of a prior stressor by exposing a separate group of observers (SW_obs_) to forced swim on day 1, and measuring their freezing to witnessing subject footshock in context B on day 2. Because freezing to footshock observation was minimal in the SW_obs_ group, observer freezing in our protocol does not appear to be a non-specific effect of heightened anxiety that results from having had a prior stressful experience. This specificity is in agreement with studies in rats and pigeons [21], [23] which suggest that prior shock selectively sensitizes emotional responding to the similar experiences of others, but not to separate forms of environmental stimuli [21], [23].

Another explanation, as previously introduced [14], is that SH_obs_ freezing may be explained by associative learning. Within our protocol, SH_obs_ may associate the unconditioned stimulus (US) of footshock with self-generated sensory cues acting as a conditioned stimulus (CS). For instance, the release of pheromones and vocalizations associated with experiencing footshock could act as a CS that become associated with the US. When observing the subject mouse experience footshock similar sensory cues produced by the subject may then serve as a CS that activates SH_obs_ own fearful associations.

According to an associative learning perspecitive, SH_obs_,freezing in our data, and observer freezing in prior papers [14], [21], [23], could also be an artifactual response to associations made between the US and other stimuli during the observer’s training. Sounds or vibrations made by the fear conditioning equipment could serve as a CS. When subsequently observing a subject being shocked, SH_obs_ freezing could be due to this CS activation of their fearful association, independent of the experience observed in the subject mouse. This explanation would be consistent with the observer requiring a similar prior experience of footshock as the subject. Contrary to this hypothesis, SH_obs_ freezing was eliminated when footshocks were delivered, but blocked from the subject with a barrier (SH_obs_-Block). Therefore, we also rule out observer freezing as an artifactual response to cues coming from the footshock equpiment.

All together, then, our experiments exclude SH_obs_ freezing as a being due to a recognition of contextual cues, a generalized sensitivity to stressors or a response to cues coming from the footshock equpiment. These data indicate that SH_obs_ freezing is specifically triggered by their recognition of a shared experience with subject mice, perhaps through an associative mechanism. Although the cues that may serve as CS within this mechanism are not known, prior studies suggest that they may reside in multisensory self-generated stimuli produced by the observer during its subjective shock experience [14], [41].

Our findings resonate with an important role for prior human experience in modulating empathy to others’ distress. In humans, the experience of prior traumatic events is associated with a greater sensitivity to others’ suffering [35]. Aversive experiences in humans facilitate their ability to recognize and share similar distressing emotions with other human beings [30], [32]–[34]. Similarly, only the SH_obs_ group, who had previously had a footshock experience, froze to witnessing a similar footshock occur to subjects.

Several other behaviors were found in the mouse that displayed similarities with human empathy. For instance, in humans indicators of emotional response initiate within seconds of observing pain in others with their magnitude predicting later pro-social behavior [39]. Similarily, with additional analysis of our data we found that SH_obs_ freezing occurred within seconds of witnessing footshock of the subject. Finally, human data show that empathy-related measures are proportional to the intensity of aversive stimuli observed or attributed to be experienced by others [34], [36]–[38]. This feature of empathy was recapitulated in our data since observer freezing increased with the number of footshocks delivered to the subject.

This study has limitations. Our assay was not sensitive to whether or not observers and subjects were cagemates or were strangers to each other. This is consistent with other studies where the social modulation of fear behavior in mice was not modified by extended periods of observer and subject cohabitation [41]. However, it remains to be determined whether observational freezing in our protocol is modified by other forms of social relation between the observer and subject. For example, other studies have detected a modulatory role when observers and subjects are siblings or are mating partners with each other [41]. We also did not investigate the influence of the estrous cycle on female behaviors [46].

In conclusion, we describe an assay for modeling several aspects of human empathy in mice. These include a role for shared prior life experience, intensity of aversive experience that observers witness occurring in others, and an acute affective response to each footshock delivered. The total assay time is two days, performed over a relatively shorter time than other methods [14], [19], [40], and delivering a relatively lower amount of aversive stimuli to mice than has been reported in other protocols [18], [19], [41]. The development of an empathy paradigm in mice will allow use of genetic tools critical for examining the neural and molecular substrates of these behaviors. The application of innovative tetracycline regulated transgenic lines and Cre/Lox systems within a mouse model of empathy, for instance, offers unique opportunities for studying brain regions, molecular players and patterns of neuronal activation that are not be accessible in rat models [47], [48]. Using this model in future studies may allow for an improved understanding of neurobiological systems for the ability to recognize and share emotion with others, a core feature of empathy that is oftentimes impaired in clinical disorders [7], [49], [50].
